# Aqua­{6,6′-dimeth­oxy-2,2′-[ethane-1,2-diylbis(nitrilo­methyl­idyne)]diphenolato}methano­lmanganese(III)) perchlorate hemihydrate

**DOI:** 10.1107/S1600536810023366

**Published:** 2010-06-26

**Authors:** Gervas Assey, Ray J. Butcher, Yilma Gultneh

**Affiliations:** a Department of Chemistry, Howard University, 525 College Street NW, Washington, DC 20059, USA

## Abstract

The asymmetric unit of the title compound, [Mn(C_18_H_18_N_2_O_4_)(CH_3_OH)(H_2_O)]ClO_4_·0.5H_2_O, contains two complex cations and two perchlorate anions, one of which is disordered over two positions in a 0.767 (8):0.233 (8) ratio. The Mn^III^ atoms are in distorted octa­hedral environments. In addition to the equatorial tetra­dentate salicylaldimine ligand, each Mn is axially coordinated by both a methanol and a water mol­ecule. The complex is a dimer held together by multiple strong and weak hydrogen-bonding inter­actions between the coordinated water mol­ecule on one monomer with all the phenolic and meth­oxy O atoms on the other monomer. In addition, the two perchlorate anions are linked by hydrogen bonds to the two methanol mol­ecules coordinated to each Mn center. The Mn—O phenolic bond distances range from 1.868 (2) to 1.882 (2) Å while the Mn—N distances range from 1.978 (2) to 1.981 (2) Å. Mn—O distances for the axial water and methanol ligands are longer at 2.226 (2)/2.257 (2) and 2.313 (2)/2.324 (2) Å, reflecting the usual Jahn–Teller distortion as found in Mn^III^ complexes.

## Related literature

For background to the use of manganese–salen compounds as single mol­ecule magnets, see: Mandal *et al.* (2009 ▶); Miyasaka *et al.* (2007 ▶); Yuan *et al.* (2007 ▶). For the use of Mn(III)–salen complexes as catalysts, see: Abashkin & Burt (2004 ▶); Chatto­padhyay *et al.* (2007 ▶); Katsuki (2000 ▶).
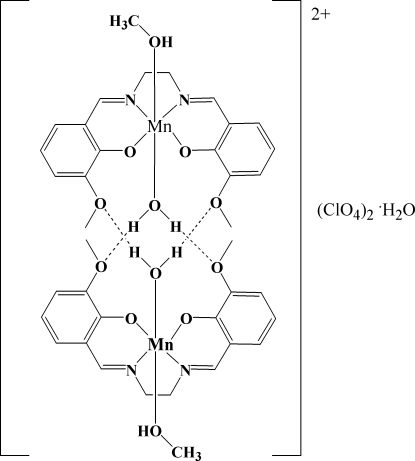


## Experimental

### 

#### Crystal data

[Mn(C_18_H_18_N_2_O_4_)(CH_4_O)(H_2_O)]ClO_4_·0.5H_2_O
*M* *_r_* = 539.80
Monoclinic, 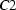

*a* = 22.7438 (15) Å
*b* = 13.3986 (9) Å
*c* = 16.3266 (10) Å
β = 101.324 (7)°
*V* = 4878.4 (5) Å^3^
*Z* = 8
Mo *K*α radiation
μ = 0.71 mm^−1^
*T* = 115 K
0.55 × 0.35 × 0.31 mm

#### Data collection

Oxford Diffraction Xcalibur diffractometer with a Ruby (Gemini Mo) detector
Absorption correction: multi-scan (*CrysAlis RED*; Oxford Diffraction, 2007 ▶) *T*_min_ = 0.610, *T*_max_ = 1.000
19303 measured reflections
13163 independent reflections
10458 reflections with *I* > 2σ(*I*)
*R*_int_ = 0.036

#### Refinement

*R*[*F* ^2^ > 2σ(*F* ^2^)] = 0.048
*wR*(*F* ^2^) = 0.127
*S* = 0.98
13163 reflections
631 parameters
55 restraints
H atoms treated by a mixture of independent and constrained refinement
Δρ_max_ = 0.74 e Å^−3^
Δρ_min_ = −0.84 e Å^−3^
Absolute structure: Flack (1983 ▶),  3800 Friedel pairs
Flack parameter: 0.254 (13)

### 

Data collection: *CrysAlis PRO* (Oxford Diffraction, 2007 ▶); cell refinement: *CrysAlis PRO*; data reduction: *CrysAlis PRO*; program(s) used to solve structure: *SHELXS97* (Sheldrick, 2008 ▶); program(s) used to refine structure: *SHELXL97* (Sheldrick, 2008 ▶); molecular graphics: *SHELXTL* (Sheldrick, 2008 ▶); software used to prepare material for publication: *SHELXTL*.

## Supplementary Material

Crystal structure: contains datablocks I, global. DOI: 10.1107/S1600536810023366/jj2037sup1.cif

Structure factors: contains datablocks I. DOI: 10.1107/S1600536810023366/jj2037Isup2.hkl

Additional supplementary materials:  crystallographic information; 3D view; checkCIF report

## Figures and Tables

**Table 1:** Hydrogen-bond geometry (Å, °)

*D*—H⋯*A*	*D*—H	H⋯*A*	*D*⋯*A*	*D*—H⋯*A*
O1*SA*—H1*SA*⋯O11	0.84	1.97	2.788 (3)	165
O1*WA*—H1*W*1⋯O3*B*	0.80 (2)	2.15 (2)	2.830 (3)	142 (3)
O1*WA*—H1*W*1⋯O1*B*	0.80 (2)	2.27 (3)	2.964 (3)	145 (3)
O1*WA*—H1*W*2⋯O4*B*	0.81 (2)	2.19 (2)	2.944 (3)	155 (3)
O1*WA*—H1*W*2⋯O2*B*	0.81 (2)	2.26 (3)	2.886 (3)	135 (3)
O1*SB*—H1*SB*⋯O21	0.84	2.02	2.737 (4)	143
O1*SB*—H1*SB*⋯O21*B*	0.84	2.33	3.010 (13)	139
O1*SB*—H1*SB*⋯O23*B*	0.84	2.50	3.312 (13)	163
O1*WB*—H1*W*3⋯O4*A*	0.82 (2)	2.23 (2)	2.944 (3)	146 (3)
O1*WB*—H1*W*3⋯O2*A*	0.82 (2)	2.19 (3)	2.885 (3)	143 (3)
O1*WB*—H1*W*4⋯O1*A*	0.83 (2)	2.12 (3)	2.868 (3)	151 (3)
O1*WB*—H1*W*4⋯O3*A*	0.83 (2)	2.36 (2)	2.997 (4)	135 (3)

**Fig. 1.**
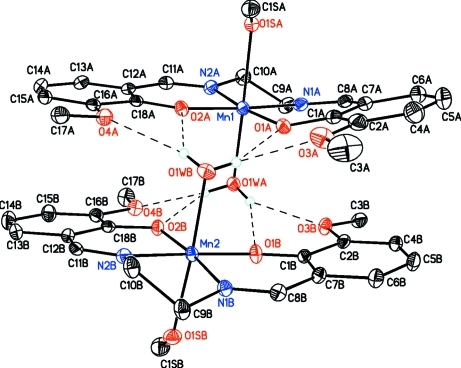
Diagram of C38H50Mn2N4O12 hydrogen bonded cation dimer, showing atom labeling. All H atoms except those attached to water are removed for clarity. Hydrogen bonds are shown by dashed lines. Thermal ellipsoids are at the 30% probability level.

**Fig. 2.**
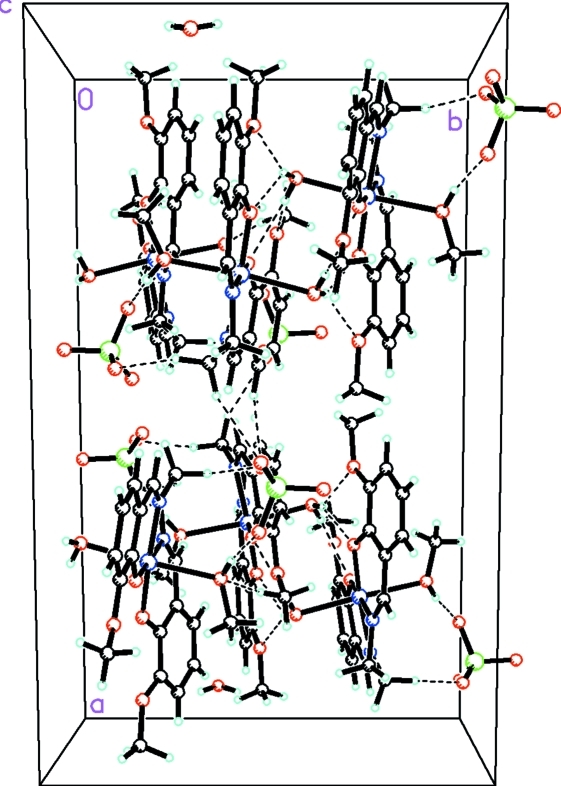
The molecular packing for C38H50Cl2Mn2N4O21 viewed down the c axis. Hydrogen bonds are shown by dashed lines.

## supplementary crystallographic information

### Comment

Small molecules of manganese(III) salen compounds are of great interest due to the fact that they can exhibit high spin complexes with S = 2 and therefore display strong magnetic uniaxial anisotropy in which the magnetic easy axis can be found as the Jahn Teller axis (Miyasaka *et al.*, 2007). They have been designed as unique magnetic systems involving molecular supermagnets such as single molecule magnets (SMM) and single chain magnets. Mandal *et al.*, (2009) synthesized a phenoxo bridged binuclear manganese(III) Schiff base complex Mn(*L*)(N3) where *L* is *N*,*N*-bis(salicyldene)-1,2-propanediamine and showed that at low temperature the complex exhibited intra-dimer ferromagnet exchange and single molecule magnet (SMM) behavior. (Yuan *et al.* (2007) conducted magnetic characterization of Mn(III) salen compounds bridged by NCNH and found that they transmit antiferromagnetic interactions between Mn(III) ions and often favored the weak ferromagnetism caused by spin canting in these one dimensional chains.

Six coordinated Mn(III)salen)OCl complex have been shown to exhibit the highest efficiency in catalyzing the epoxidation reaction irrespective of oxidant and solvent used (Chattopadhyay *et al.*, 2007). Mn(III) salen compounds have proved to be promising as synthetic antioxidants, in particular dismutation of H_2_O_2_ resulting into 2 water molecules and oxygen and they are used to study the catalytic mechanism of the functional biomimetic enzymes (Abashkin & Burt, 2004). Achiral salen manganese complexes have been used as catalysts for asymmetric epoxidation (Katsuki, 2000).

In the title compound, C_38_H_50_Cl_2_Mn_2_N_4_O_21_, each Mn is in a distorted octahedral environment. Each metal ion in the complex is coordinated to both a methanol and a water molecule. The complex is a dimer held to together by multiple strong and weak hydrogen bonding interactions between the coordinated water molecule on the other monomer with all the phenolic and methoxy oxygen atoms on the other monomer. In addition the two perchlorate anions are linked by hydrogen bonds to the two methanol molecules coordinated to each Mn center. The Mn—O phenolic bond distances range from 1.868 (2) to 1.882 (2) Å while the Mn—N distances range from 1.978 (2) to 1.981 (2) Å. Mn—O distances for the axial water and methanol ligands are longer at 2.226 (2)/2.257 (2) and 2.313 (2)/2.324 (2) Å reflecting the usual Jahn Teller distortion as found in Mn(III) complexes.

### Experimental

The synthesis of the ligand, ethylenebis(3-methoxysalicylaldimine) was achieved by adding a solution of *o*-vanillin in 40 ml me thanol dropwise using glass pipette to a solution of (2 g, 33.3 mmol) of ethylenediamine in 10 ml me thanol. The mixture was refluxed for 24 h. After solvent evaporation under reduced pressure, yellow solids were obtained.

Synthesis of the complex C_38_H_50_Cl_2_Mn_2_N4_O_21 was achieved by adding to a solution of (0.36 g, 1 mmol) of Mn(ClO_4_)_2_.6H_2_O in 5 ml me thanol a solution of (0.33 g, 1 mmol) H_2_*L*_2_ in 3 ml of dichloromethane. The mixture was stirred at room temperature for 1 h. The mixture was then filtered and layered with diethyl ether for crystallization. Crystals suitable for X-ray analysis were obtained.

### Refinement

H atoms were placed in geometrically idealized positions and constrained to ride on their parent atoms with a C—H distances of 0.95 and 0.99 Å *U*_iso_(H) = 1.2*U*_eq_(C) and 0.98 Å for CH_3_ [*U*_iso_(H) = 1.5*U*_eq_(C)]. Water H atoms were refined isotropically with O—H distance restrained to 0.82 Å and H—O—H angle to 104.5° with [*U*_iso_(H) = 1.5*U*_eq_(O)]. The structure contained disordered water molecules near symmetry elements. These were removed using the SQUEEZE routine in *PLATON* (Spek, 2009).

### Crystal data

**Table:**

[Mn(C_18_H_18_N_2_O_4_)(CH_4_O)(H_2_O)]ClO_4_·0.5H_2_O	*F*(000) = 2232
*M**_r_* = 539.80	*D*_x_ = 1.470 Mg m^−^^3^
Monoclinic, *C*2	Mo *K*α radiation, λ = 0.71073 Å
Hall symbol: C 2y	Cell parameters from 7290 reflections
*a* = 22.7438 (15) Å	θ = 4.7–32.7°
*b* = 13.3986 (9) Å	µ = 0.71 mm^−^^1^
*c* = 16.3266 (10) Å	*T* = 115 K
β = 101.324 (7)°	Prism, black
*V* = 4878.4 (5) Å^3^	0.55 × 0.35 × 0.31 mm
*Z* = 8	

### Data collection

**Table:**

Oxford Diffraction Xcalibur diffractometer with a Ruby (Gemini Mo) detector	13163 independent reflections
Radiation source: Enhance (Mo) X-ray Source	10458 reflections with *I* > 2σ(*I*)
graphite	*R*_int_ = 0.036
Detector resolution: 10.5081 pixels mm^-1^	θ_max_ = 32.8°, θ_min_ = 4.7°
ω scans	*h* = −20→33
Absorption correction: multi-scan (*CrysAlis RED*; Oxford Diffraction, 2007)	*k* = −20→19
*T*_min_ = 0.610, *T*_max_ = 1.000	*l* = −23→24
19303 measured reflections	

### Refinement

**Table:**

Refinement on *F*^2^	Secondary atom site location: difference Fourier map
Least-squares matrix: full	Hydrogen site location: inferred from neighbouring sites
*R*[*F*^2^ > 2σ(*F*^2^)] = 0.048	H atoms treated by a mixture of independent and constrained refinement
*wR*(*F*^2^) = 0.127	*w* = 1/[σ^2^(*F*_o_^2^) + (0.0828*P*)^2^] where *P* = (*F*_o_^2^ + 2*F*_c_^2^)/3
*S* = 0.98	(Δ/σ)_max_ = 0.001
13163 reflections	Δρ_max_ = 0.74 e Å^−^^3^
631 parameters	Δρ_min_ = −0.84 e Å^−^^3^
55 restraints	Absolute structure: Flack (1983), 3800 Friedel pairs
Primary atom site location: structure-invariant direct methods	Flack parameter: 0.254 (13)

### Special details

**Table:**

Geometry. All e.s.d.'s (except the e.s.d. in the dihedral angle between two l.s. planes) are estimated using the full covariance matrix. The cell e.s.d.'s are taken into account individually in the estimation of e.s.d.'s in distances, angles and torsion angles; correlations between e.s.d.'s in cell parameters are only used when they are defined by crystal symmetry. An approximate (isotropic) treatment of cell e.s.d.'s is used for estimating e.s.d.'s involving l.s. planes.
Refinement. Refinement of *F*^2^ against ALL reflections. The weighted *R*-factor *wR* and goodness of fit *S* are based on *F*^2^, conventional *R*-factors *R* are based on *F*, with *F* set to zero for negative *F*^2^. The threshold expression of *F*^2^ > σ(*F*^2^) is used only for calculating *R*-factors(gt) *etc*. and is not relevant to the choice of reflections for refinement. *R*-factors based on *F*^2^ are statistically about twice as large as those based on *F*, and *R*- factors based on ALL data will be even larger.

### Fractional atomic coordinates and isotropic or equivalent isotropic displacement parameters (Å^2^)

**Table:**

	*x*	*y*	*z*	*U*_iso_*/*U*_eq_	Occ. (<1)
Mn1	0.284266 (19)	0.22062 (6)	0.31977 (3)	0.02020 (10)	
O1A	0.20506 (9)	0.20431 (17)	0.33561 (13)	0.0238 (4)	
O2A	0.26446 (9)	0.19945 (17)	0.20359 (12)	0.0230 (4)	
O3A	0.08934 (11)	0.2039 (2)	0.31066 (19)	0.0413 (6)	
O4A	0.21244 (11)	0.16204 (18)	0.05267 (13)	0.0287 (5)	
O1SA	0.26218 (9)	0.38821 (17)	0.29203 (13)	0.0246 (4)	
H1SA	0.2885	0.4128	0.2683	0.030*	
O1WA	0.30967 (9)	0.06162 (17)	0.34674 (13)	0.0228 (4)	
H1W1	0.2831 (12)	0.0331 (15)	0.363 (2)	0.034*	
H1W2	0.3150 (16)	0.0339 (16)	0.3049 (15)	0.034*	
N1A	0.31187 (12)	0.2484 (2)	0.44013 (15)	0.0255 (5)	
N2A	0.36935 (11)	0.2478 (2)	0.31556 (15)	0.0232 (5)	
C1A	0.18088 (15)	0.2407 (2)	0.3969 (2)	0.0268 (6)	
C2A	0.11764 (16)	0.2419 (3)	0.3859 (3)	0.0359 (8)	
C3A	0.02547 (17)	0.1982 (4)	0.2937 (4)	0.0614 (14)	
H3AA	0.0124	0.1557	0.3357	0.092*	
H3AB	0.0115	0.1696	0.2381	0.092*	
H3AC	0.0087	0.2652	0.2959	0.092*	
C4A	0.08952 (19)	0.2788 (3)	0.4481 (3)	0.0477 (10)	
H4AA	0.0470	0.2798	0.4396	0.057*	
C5A	0.1234 (2)	0.3140 (4)	0.5226 (3)	0.0572 (13)	
H5AA	0.1040	0.3384	0.5650	0.069*	
C6A	0.1845 (2)	0.3138 (3)	0.5352 (2)	0.0429 (9)	
H6AA	0.2072	0.3389	0.5862	0.051*	
C7A	0.21462 (16)	0.2769 (2)	0.4740 (2)	0.0303 (7)	
C8A	0.27886 (17)	0.2726 (2)	0.4925 (2)	0.0294 (7)	
H8AA	0.2985	0.2888	0.5477	0.035*	
C9A	0.37745 (15)	0.2394 (3)	0.46612 (19)	0.0315 (7)	
H9AA	0.3889	0.1686	0.4764	0.038*	
H9AB	0.3916	0.2775	0.5183	0.038*	
C10A	0.40551 (15)	0.2815 (3)	0.39549 (19)	0.0302 (7)	
H10C	0.4062	0.3553	0.3979	0.036*	
H10D	0.4473	0.2574	0.4012	0.036*	
C11A	0.39377 (14)	0.2389 (2)	0.25053 (19)	0.0239 (6)	
H11B	0.4358	0.2498	0.2582	0.029*	
C12A	0.36241 (14)	0.2138 (2)	0.16743 (18)	0.0243 (6)	
C13A	0.39642 (16)	0.2077 (3)	0.1042 (2)	0.0304 (7)	
H13A	0.4386	0.2172	0.1178	0.037*	
C14A	0.36904 (17)	0.1882 (3)	0.0230 (2)	0.0336 (7)	
H14B	0.3920	0.1855	−0.0196	0.040*	
C15A	0.30703 (18)	0.1723 (3)	0.00364 (19)	0.0326 (8)	
H15B	0.2882	0.1584	−0.0524	0.039*	
C16A	0.27278 (15)	0.1765 (2)	0.06459 (18)	0.0258 (6)	
C17A	0.18066 (17)	0.1551 (3)	−0.0314 (2)	0.0335 (7)	
H17A	0.1841	0.2184	−0.0602	0.050*	
H17B	0.1383	0.1409	−0.0320	0.050*	
H17C	0.1977	0.1012	−0.0600	0.050*	
C18A	0.30015 (14)	0.1966 (2)	0.14890 (17)	0.0206 (5)	
C1SA	0.20369 (15)	0.4126 (3)	0.2451 (2)	0.0328 (7)	
H1SC	0.2047	0.4791	0.2204	0.049*	
H1SD	0.1747	0.4121	0.2823	0.049*	
H1SE	0.1918	0.3633	0.2006	0.049*	
Mn2	0.175942 (18)	−0.06031 (6)	0.20590 (2)	0.01856 (9)	
O1B	0.19422 (8)	−0.04764 (19)	0.32295 (12)	0.0240 (4)	
O2B	0.25505 (9)	−0.03912 (17)	0.19454 (12)	0.0214 (4)	
O3B	0.24161 (9)	0.01694 (19)	0.46979 (12)	0.0247 (4)	
O4B	0.36976 (9)	−0.03079 (18)	0.22170 (13)	0.0252 (4)	
O1SB	0.19546 (10)	−0.22959 (18)	0.21297 (15)	0.0295 (5)	
H1SB	0.1695	−0.2683	0.2252	0.035*	
O1WB	0.14964 (10)	0.10166 (17)	0.18614 (14)	0.0271 (5)	
H1W3	0.1767 (13)	0.1277 (16)	0.167 (2)	0.041*	
H1W4	0.1538 (16)	0.1265 (16)	0.2332 (13)	0.041*	
N1B	0.09088 (10)	−0.0906 (2)	0.20692 (15)	0.0208 (5)	
N2B	0.14853 (10)	−0.0746 (2)	0.08354 (14)	0.0195 (5)	
C1B	0.15614 (13)	−0.0289 (2)	0.37347 (17)	0.0191 (5)	
C2B	0.18095 (13)	0.0050 (2)	0.45525 (16)	0.0194 (5)	
C3B	0.27128 (14)	0.0354 (3)	0.55419 (18)	0.0272 (6)	
H3BA	0.2585	0.1002	0.5724	0.041*	
H3BB	0.3148	0.0361	0.5574	0.041*	
H3BC	0.2609	−0.0173	0.5905	0.041*	
C4B	0.14463 (14)	0.0239 (2)	0.51249 (18)	0.0254 (6)	
H4BA	0.1617	0.0470	0.5670	0.030*	
C5B	0.08227 (14)	0.0086 (3)	0.48935 (19)	0.0283 (6)	
H5BA	0.0572	0.0218	0.5282	0.034*	
C6B	0.05785 (14)	−0.0251 (3)	0.41128 (19)	0.0262 (6)	
H6BA	0.0158	−0.0357	0.3965	0.031*	
C7B	0.09403 (12)	−0.0446 (2)	0.35185 (16)	0.0210 (5)	
C8B	0.06494 (12)	−0.0778 (2)	0.26962 (18)	0.0221 (6)	
H8BA	0.0232	−0.0913	0.2610	0.027*	
C9B	0.05733 (13)	−0.1273 (3)	0.12582 (18)	0.0256 (6)	
H9BA	0.0141	−0.1124	0.1200	0.031*	
H9BB	0.0624	−0.2003	0.1214	0.031*	
C10B	0.08281 (12)	−0.0736 (3)	0.05825 (17)	0.0259 (6)	
H10A	0.0703	−0.1079	0.0039	0.031*	
H10B	0.0680	−0.0040	0.0523	0.031*	
C11B	0.18300 (13)	−0.0831 (2)	0.02926 (17)	0.0203 (5)	
H11A	0.1642	−0.0935	−0.0274	0.024*	
C12B	0.24705 (13)	−0.0780 (2)	0.04822 (17)	0.0203 (5)	
C13B	0.27827 (15)	−0.0926 (3)	−0.01842 (19)	0.0265 (6)	
H13B	0.2563	−0.1053	−0.0732	0.032*	
C14B	0.33933 (15)	−0.0886 (3)	−0.0047 (2)	0.0321 (7)	
H14A	0.3597	−0.0983	−0.0496	0.038*	
C15B	0.37194 (14)	−0.0700 (3)	0.07584 (19)	0.0288 (6)	
H15A	0.4145	−0.0693	0.0857	0.035*	
C16B	0.34283 (12)	−0.0528 (2)	0.14082 (17)	0.0220 (5)	
C17B	0.43359 (14)	−0.0242 (3)	0.2403 (2)	0.0343 (7)	
H17D	0.4508	−0.0870	0.2251	0.051*	
H17E	0.4473	−0.0118	0.3001	0.051*	
H17F	0.4466	0.0307	0.2083	0.051*	
C18B	0.27935 (12)	−0.0572 (2)	0.12807 (16)	0.0192 (5)	
C1SB	0.25317 (16)	−0.2747 (3)	0.2351 (2)	0.0356 (7)	
H2SA	0.2832	−0.2312	0.2179	0.053*	
H2SB	0.2528	−0.3394	0.2069	0.053*	
H2SC	0.2632	−0.2846	0.2957	0.053*	
Cl1	0.39136 (3)	0.51826 (7)	0.21382 (4)	0.02536 (15)	
O11	0.33111 (11)	0.4789 (2)	0.18945 (15)	0.0389 (6)	
O12	0.38896 (13)	0.6234 (2)	0.21341 (19)	0.0485 (7)	
O13	0.41788 (12)	0.4822 (2)	0.29518 (15)	0.0438 (7)	
O14	0.42573 (13)	0.4846 (3)	0.15440 (19)	0.0575 (9)	
Cl2	0.07547 (4)	−0.35939 (7)	0.29865 (7)	0.0554 (3)	
O21	0.13420 (10)	−0.3178 (3)	0.3221 (2)	0.0422 (9)	0.767 (8)
O22	0.07724 (17)	−0.46343 (15)	0.3180 (3)	0.0489 (11)	0.767 (8)
O23	0.0539 (2)	−0.3456 (4)	0.21119 (14)	0.107 (2)	0.767 (8)
O24	0.03594 (15)	−0.3100 (3)	0.3440 (3)	0.0821 (18)	0.767 (8)
O21B	0.1283 (3)	−0.3092 (9)	0.3406 (7)	0.0422 (9)	0.233 (8)
O22B	0.0679 (6)	−0.4497 (6)	0.3420 (8)	0.0489 (11)	0.233 (8)
O23B	0.0813 (7)	−0.3824 (11)	0.2151 (4)	0.107 (2)	0.233 (8)
O24B	0.0246 (4)	−0.2964 (7)	0.2968 (10)	0.0821 (18)	0.233 (8)

### Atomic displacement parameters (Å^2^)

**Table:**

	*U*^11^	*U*^22^	*U*^33^	*U*^12^	*U*^13^	*U*^23^
Mn1	0.0235 (2)	0.0206 (2)	0.01571 (18)	−0.00317 (17)	0.00203 (15)	−0.00269 (17)
O1A	0.0259 (10)	0.0225 (11)	0.0236 (10)	−0.0017 (9)	0.0066 (8)	−0.0033 (9)
O2A	0.0262 (10)	0.0238 (11)	0.0177 (9)	−0.0024 (8)	0.0008 (7)	−0.0059 (8)
O3A	0.0260 (11)	0.0359 (14)	0.0597 (17)	0.0011 (10)	0.0026 (11)	−0.0045 (13)
O4A	0.0384 (12)	0.0271 (11)	0.0172 (9)	−0.0029 (10)	−0.0027 (8)	0.0002 (9)
O1SA	0.0272 (11)	0.0234 (10)	0.0238 (10)	0.0006 (9)	0.0062 (8)	−0.0005 (9)
O1WA	0.0257 (10)	0.0239 (10)	0.0190 (10)	−0.0047 (8)	0.0045 (8)	−0.0032 (8)
N1A	0.0340 (14)	0.0223 (12)	0.0188 (11)	−0.0074 (11)	0.0020 (10)	−0.0003 (10)
N2A	0.0252 (12)	0.0254 (12)	0.0172 (10)	−0.0043 (10)	−0.0001 (9)	−0.0010 (10)
C1A	0.0337 (16)	0.0189 (14)	0.0303 (15)	0.0022 (12)	0.0125 (12)	0.0041 (12)
C2A	0.0378 (18)	0.0248 (16)	0.048 (2)	0.0071 (14)	0.0169 (15)	0.0058 (15)
C3A	0.0229 (17)	0.057 (3)	0.100 (4)	0.0107 (18)	0.000 (2)	0.012 (3)
C4A	0.045 (2)	0.041 (2)	0.065 (3)	0.0127 (18)	0.030 (2)	0.006 (2)
C5A	0.081 (3)	0.043 (2)	0.062 (3)	0.008 (2)	0.050 (3)	0.002 (2)
C6A	0.072 (3)	0.0278 (16)	0.0364 (19)	0.0006 (18)	0.0280 (18)	−0.0016 (15)
C7A	0.0475 (19)	0.0191 (13)	0.0274 (15)	−0.0011 (14)	0.0146 (14)	0.0020 (13)
C8A	0.0503 (19)	0.0198 (14)	0.0188 (13)	−0.0080 (14)	0.0083 (13)	−0.0005 (12)
C9A	0.0364 (17)	0.0347 (18)	0.0202 (14)	−0.0083 (14)	−0.0019 (12)	0.0038 (14)
C10A	0.0325 (16)	0.0335 (16)	0.0219 (14)	−0.0124 (14)	−0.0011 (12)	−0.0043 (13)
C11A	0.0240 (13)	0.0215 (14)	0.0258 (14)	−0.0002 (11)	0.0040 (11)	0.0023 (12)
C12A	0.0344 (15)	0.0161 (12)	0.0242 (13)	0.0003 (12)	0.0099 (11)	0.0033 (12)
C13A	0.0411 (18)	0.0223 (14)	0.0304 (15)	−0.0038 (14)	0.0132 (13)	0.0022 (13)
C14A	0.054 (2)	0.0261 (16)	0.0248 (15)	−0.0045 (15)	0.0169 (14)	0.0035 (13)
C15A	0.061 (2)	0.0207 (14)	0.0153 (13)	−0.0006 (15)	0.0054 (13)	−0.0005 (12)
C16A	0.0434 (17)	0.0143 (12)	0.0186 (13)	−0.0001 (12)	0.0034 (12)	0.0005 (11)
C17A	0.051 (2)	0.0261 (16)	0.0186 (14)	−0.0049 (15)	−0.0060 (13)	−0.0020 (12)
C18A	0.0324 (14)	0.0127 (12)	0.0154 (11)	−0.0010 (11)	0.0011 (10)	−0.0005 (10)
C1SA	0.0329 (16)	0.0329 (17)	0.0312 (16)	0.0044 (14)	0.0024 (12)	0.0033 (14)
Mn2	0.01845 (18)	0.0221 (2)	0.01472 (18)	−0.00026 (17)	0.00234 (13)	−0.00233 (17)
O1B	0.0199 (9)	0.0345 (13)	0.0179 (9)	−0.0008 (9)	0.0049 (7)	−0.0054 (9)
O2B	0.0218 (9)	0.0262 (11)	0.0168 (9)	−0.0032 (8)	0.0051 (7)	−0.0021 (8)
O3B	0.0241 (10)	0.0340 (12)	0.0152 (9)	−0.0039 (9)	0.0019 (7)	−0.0006 (9)
O4B	0.0186 (9)	0.0295 (11)	0.0263 (10)	0.0016 (8)	0.0017 (7)	0.0007 (9)
O1SB	0.0304 (11)	0.0217 (11)	0.0371 (12)	0.0018 (9)	0.0087 (9)	0.0017 (10)
O1WB	0.0296 (11)	0.0216 (10)	0.0295 (11)	−0.0024 (9)	0.0043 (9)	−0.0035 (9)
N1B	0.0207 (11)	0.0223 (11)	0.0184 (11)	−0.0001 (9)	0.0013 (8)	−0.0026 (9)
N2B	0.0198 (10)	0.0207 (12)	0.0167 (10)	0.0001 (9)	0.0000 (8)	0.0008 (9)
C1B	0.0236 (13)	0.0177 (12)	0.0173 (12)	0.0036 (10)	0.0071 (10)	0.0024 (10)
C2B	0.0250 (13)	0.0201 (13)	0.0137 (11)	−0.0014 (11)	0.0050 (9)	0.0023 (10)
C3B	0.0345 (16)	0.0268 (15)	0.0182 (13)	−0.0054 (13)	−0.0003 (11)	0.0024 (12)
C4B	0.0369 (16)	0.0237 (14)	0.0172 (12)	−0.0030 (13)	0.0092 (11)	−0.0007 (12)
C5B	0.0317 (15)	0.0334 (16)	0.0236 (14)	0.0019 (13)	0.0148 (11)	0.0016 (13)
C6B	0.0230 (13)	0.0329 (16)	0.0242 (13)	−0.0001 (12)	0.0083 (11)	−0.0019 (13)
C7B	0.0225 (12)	0.0242 (14)	0.0163 (11)	−0.0011 (11)	0.0039 (9)	0.0016 (11)
C8B	0.0190 (12)	0.0232 (15)	0.0243 (13)	0.0022 (11)	0.0047 (10)	0.0011 (12)
C9B	0.0192 (13)	0.0358 (17)	0.0206 (13)	−0.0048 (12)	0.0015 (10)	−0.0087 (13)
C10B	0.0207 (12)	0.0360 (17)	0.0187 (12)	−0.0003 (13)	−0.0017 (9)	−0.0059 (13)
C11B	0.0280 (13)	0.0168 (12)	0.0152 (11)	−0.0039 (11)	0.0020 (10)	−0.0024 (10)
C12B	0.0248 (13)	0.0192 (13)	0.0175 (11)	−0.0002 (11)	0.0055 (9)	0.0000 (11)
C13B	0.0355 (16)	0.0258 (14)	0.0195 (13)	−0.0021 (13)	0.0083 (11)	−0.0034 (12)
C14B	0.0342 (16)	0.0383 (18)	0.0256 (15)	0.0035 (14)	0.0107 (12)	−0.0016 (14)
C15B	0.0261 (14)	0.0341 (17)	0.0272 (14)	0.0035 (14)	0.0078 (11)	−0.0022 (14)
C16B	0.0238 (12)	0.0200 (13)	0.0222 (12)	0.0009 (12)	0.0046 (10)	−0.0007 (12)
C17B	0.0250 (15)	0.045 (2)	0.0304 (16)	0.0016 (14)	0.0010 (12)	0.0012 (15)
C18B	0.0221 (11)	0.0149 (11)	0.0204 (11)	−0.0012 (11)	0.0036 (9)	0.0014 (11)
C1SB	0.0391 (17)	0.0331 (17)	0.0341 (16)	0.0072 (15)	0.0061 (13)	0.0076 (15)
Cl1	0.0251 (3)	0.0284 (3)	0.0208 (3)	−0.0007 (3)	0.0000 (2)	0.0038 (3)
O11	0.0317 (12)	0.0508 (16)	0.0313 (12)	−0.0159 (11)	−0.0005 (9)	0.0119 (11)
O12	0.0504 (16)	0.0237 (12)	0.0629 (18)	−0.0003 (12)	−0.0096 (14)	0.0024 (13)
O13	0.0422 (14)	0.0570 (17)	0.0265 (12)	−0.0075 (13)	−0.0074 (10)	0.0143 (12)
O14	0.0433 (15)	0.085 (3)	0.0461 (16)	0.0162 (16)	0.0144 (12)	−0.0123 (16)
Cl2	0.0366 (5)	0.0504 (6)	0.0679 (7)	−0.0131 (4)	−0.0175 (4)	0.0301 (6)
O21	0.0329 (14)	0.063 (2)	0.029 (2)	−0.0123 (14)	0.0022 (12)	0.0160 (16)
O22	0.043 (2)	0.0374 (18)	0.070 (3)	0.0021 (15)	0.0215 (18)	0.0097 (19)
O23	0.096 (4)	0.117 (4)	0.078 (3)	−0.050 (3)	−0.058 (3)	0.057 (3)
O24	0.0325 (19)	0.074 (3)	0.125 (5)	0.0189 (19)	−0.019 (2)	−0.004 (3)
O21B	0.0329 (14)	0.063 (2)	0.029 (2)	−0.0123 (14)	0.0022 (12)	0.0160 (16)
O22B	0.043 (2)	0.0374 (18)	0.070 (3)	0.0021 (15)	0.0215 (18)	0.0097 (19)
O23B	0.096 (4)	0.117 (4)	0.078 (3)	−0.050 (3)	−0.058 (3)	0.057 (3)
O24B	0.0325 (19)	0.074 (3)	0.125 (5)	0.0189 (19)	−0.019 (2)	−0.004 (3)

### Geometric parameters (Å, °)

**Table:**

Mn1—O1A	1.882 (2)	O1B—C1B	1.332 (3)
Mn1—O2A	1.883 (2)	O2B—C18B	1.333 (3)
Mn1—N1A	1.977 (3)	O3B—C2B	1.363 (3)
Mn1—N2A	1.983 (3)	O3B—C3B	1.432 (3)
Mn1—O1WA	2.229 (2)	O4B—C16B	1.374 (4)
Mn1—O1SA	2.326 (2)	O4B—C17B	1.426 (4)
O1A—C1A	1.325 (4)	O1SB—C1SB	1.426 (4)
O2A—C18A	1.320 (4)	O1SB—H1SB	0.8400
O3A—C2A	1.369 (5)	O1WB—H1W3	0.821 (17)
O3A—C3A	1.427 (4)	O1WB—H1W4	0.826 (17)
O4A—C16A	1.362 (4)	N1B—C8B	1.289 (4)
O4A—C17A	1.424 (4)	N1B—C9B	1.476 (4)
O1SA—C1SA	1.437 (4)	N2B—C11B	1.298 (3)
O1SA—H1SA	0.8400	N2B—C10B	1.470 (3)
O1WA—H1W1	0.802 (17)	C1B—C7B	1.403 (4)
O1WA—H1W2	0.807 (17)	C1B—C2B	1.418 (4)
N1A—C8A	1.285 (4)	C2B—C4B	1.386 (4)
N1A—C9A	1.473 (4)	C3B—H3BA	0.9800
N2A—C11A	1.297 (4)	C3B—H3BB	0.9800
N2A—C10A	1.470 (4)	C3B—H3BC	0.9800
C1A—C2A	1.414 (5)	C4B—C5B	1.409 (4)
C1A—C7A	1.425 (5)	C4B—H4BA	0.9500
C2A—C4A	1.393 (5)	C5B—C6B	1.363 (4)
C3A—H3AA	0.9800	C5B—H5BA	0.9500
C3A—H3AB	0.9800	C6B—C7B	1.414 (4)
C3A—H3AC	0.9800	C6B—H6BA	0.9500
C4A—C5A	1.389 (7)	C7B—C8B	1.445 (4)
C4A—H4AA	0.9500	C8B—H8BA	0.9500
C5A—C6A	1.364 (6)	C9B—C10B	1.524 (4)
C5A—H5AA	0.9500	C9B—H9BA	0.9900
C6A—C7A	1.408 (5)	C9B—H9BB	0.9900
C6A—H6AA	0.9500	C10B—H10A	0.9900
C7A—C8A	1.434 (5)	C10B—H10B	0.9900
C8A—H8AA	0.9500	C11B—C12B	1.430 (4)
C9A—C10A	1.531 (5)	C11B—H11A	0.9500
C9A—H9AA	0.9900	C12B—C18B	1.393 (4)
C9A—H9AB	0.9900	C12B—C13B	1.425 (4)
C10A—H10C	0.9900	C13B—C14B	1.364 (5)
C10A—H10D	0.9900	C13B—H13B	0.9500
C11A—C12A	1.443 (4)	C14B—C15B	1.399 (5)
C11A—H11B	0.9500	C14B—H14A	0.9500
C12A—C18A	1.407 (4)	C15B—C16B	1.376 (4)
C12A—C13A	1.409 (4)	C15B—H15A	0.9500
C13A—C14A	1.375 (5)	C16B—C18B	1.419 (4)
C13A—H13A	0.9500	C17B—H17D	0.9800
C14A—C15A	1.400 (5)	C17B—H17E	0.9800
C14A—H14B	0.9500	C17B—H17F	0.9800
C15A—C16A	1.380 (5)	C1SB—H2SA	0.9800
C15A—H15B	0.9500	C1SB—H2SB	0.9800
C16A—C18A	1.421 (4)	C1SB—H2SC	0.9800
C17A—H17A	0.9800	Cl1—O12	1.410 (3)
C17A—H17B	0.9800	Cl1—O13	1.431 (2)
C17A—H17C	0.9800	Cl1—O14	1.433 (3)
C1SA—H1SC	0.9800	Cl1—O11	1.449 (2)
C1SA—H1SD	0.9800	Cl2—O23	1.4279 (19)
C1SA—H1SE	0.9800	Cl2—O22	1.4281 (19)
Mn2—O2B	1.866 (2)	Cl2—O21	1.4284 (19)
Mn2—O1B	1.8819 (19)	Cl2—O24B	1.429 (2)
Mn2—N1B	1.980 (2)	Cl2—O22B	1.4291 (19)
Mn2—N2B	1.982 (2)	Cl2—O21B	1.4299 (19)
Mn2—O1WB	2.257 (2)	Cl2—O23B	1.4303 (19)
Mn2—O1SB	2.310 (2)	Cl2—O24	1.4339 (19)
			
O1A—Mn1—O2A	94.29 (9)	C2B—O3B—C3B	117.2 (2)
O1A—Mn1—N1A	90.63 (10)	C16B—O4B—C17B	117.4 (2)
O2A—Mn1—N1A	174.83 (10)	C1SB—O1SB—Mn2	126.0 (2)
O1A—Mn1—N2A	173.05 (10)	C1SB—O1SB—H1SB	109.5
O2A—Mn1—N2A	91.99 (10)	Mn2—O1SB—H1SB	118.8
N1A—Mn1—N2A	83.01 (11)	Mn2—O1WB—H1W3	105.4 (17)
O1A—Mn1—O1WA	94.57 (9)	Mn2—O1WB—H1W4	106.0 (17)
O2A—Mn1—O1WA	93.24 (9)	H1W3—O1WB—H1W4	103 (2)
N1A—Mn1—O1WA	87.93 (10)	C8B—N1B—C9B	121.3 (2)
N2A—Mn1—O1WA	88.02 (9)	C8B—N1B—Mn2	125.4 (2)
O1A—Mn1—O1SA	87.88 (9)	C9B—N1B—Mn2	113.24 (18)
O2A—Mn1—O1SA	87.11 (9)	C11B—N2B—C10B	121.7 (2)
N1A—Mn1—O1SA	91.51 (10)	C11B—N2B—Mn2	125.72 (19)
N2A—Mn1—O1SA	89.48 (9)	C10B—N2B—Mn2	112.54 (17)
O1WA—Mn1—O1SA	177.49 (8)	O1B—C1B—C7B	124.1 (3)
C1A—O1A—Mn1	127.7 (2)	O1B—C1B—C2B	117.2 (2)
C18A—O2A—Mn1	128.94 (19)	C7B—C1B—C2B	118.7 (2)
C2A—O3A—C3A	118.4 (3)	O3B—C2B—C4B	125.3 (2)
C16A—O4A—C17A	117.0 (3)	O3B—C2B—C1B	113.8 (2)
C1SA—O1SA—Mn1	117.75 (19)	C4B—C2B—C1B	120.9 (3)
C1SA—O1SA—H1SA	109.5	O3B—C3B—H3BA	109.5
Mn1—O1SA—H1SA	108.8	O3B—C3B—H3BB	109.5
Mn1—O1WA—H1W1	109.7 (17)	H3BA—C3B—H3BB	109.5
Mn1—O1WA—H1W2	110.4 (17)	O3B—C3B—H3BC	109.5
H1W1—O1WA—H1W2	108 (3)	H3BA—C3B—H3BC	109.5
C8A—N1A—C9A	121.3 (3)	H3BB—C3B—H3BC	109.5
C8A—N1A—Mn1	126.5 (2)	C2B—C4B—C5B	119.6 (3)
C9A—N1A—Mn1	112.1 (2)	C2B—C4B—H4BA	120.2
C11A—N2A—C10A	120.0 (3)	C5B—C4B—H4BA	120.2
C11A—N2A—Mn1	126.1 (2)	C6B—C5B—C4B	120.2 (3)
C10A—N2A—Mn1	113.9 (2)	C6B—C5B—H5BA	119.9
O1A—C1A—C2A	118.1 (3)	C4B—C5B—H5BA	119.9
O1A—C1A—C7A	124.1 (3)	C5B—C6B—C7B	121.1 (3)
C2A—C1A—C7A	117.7 (3)	C5B—C6B—H6BA	119.4
O3A—C2A—C4A	125.8 (3)	C7B—C6B—H6BA	119.4
O3A—C2A—C1A	113.3 (3)	C1B—C7B—C6B	119.5 (3)
C4A—C2A—C1A	120.9 (4)	C1B—C7B—C8B	122.3 (2)
O3A—C3A—H3AA	109.5	C6B—C7B—C8B	118.2 (3)
O3A—C3A—H3AB	109.5	N1B—C8B—C7B	125.5 (3)
H3AA—C3A—H3AB	109.5	N1B—C8B—H8BA	117.2
O3A—C3A—H3AC	109.5	C7B—C8B—H8BA	117.2
H3AA—C3A—H3AC	109.5	N1B—C9B—C10B	106.8 (2)
H3AB—C3A—H3AC	109.5	N1B—C9B—H9BA	110.4
C5A—C4A—C2A	120.2 (4)	C10B—C9B—H9BA	110.4
C5A—C4A—H4AA	119.9	N1B—C9B—H9BB	110.4
C2A—C4A—H4AA	119.9	C10B—C9B—H9BB	110.4
C6A—C5A—C4A	120.4 (4)	H9BA—C9B—H9BB	108.6
C6A—C5A—H5AA	119.8	N2B—C10B—C9B	107.6 (2)
C4A—C5A—H5AA	119.8	N2B—C10B—H10A	110.2
C5A—C6A—C7A	121.1 (4)	C9B—C10B—H10A	110.2
C5A—C6A—H6AA	119.5	N2B—C10B—H10B	110.2
C7A—C6A—H6AA	119.5	C9B—C10B—H10B	110.2
C6A—C7A—C1A	119.7 (3)	H10A—C10B—H10B	108.5
C6A—C7A—C8A	118.8 (3)	N2B—C11B—C12B	125.0 (2)
C1A—C7A—C8A	121.5 (3)	N2B—C11B—H11A	117.5
N1A—C8A—C7A	125.1 (3)	C12B—C11B—H11A	117.5
N1A—C8A—H8AA	117.5	C18B—C12B—C13B	119.4 (3)
C7A—C8A—H8AA	117.5	C18B—C12B—C11B	122.7 (2)
N1A—C9A—C10A	107.5 (3)	C13B—C12B—C11B	117.9 (3)
N1A—C9A—H9AA	110.2	C14B—C13B—C12B	120.9 (3)
C10A—C9A—H9AA	110.2	C14B—C13B—H13B	119.6
N1A—C9A—H9AB	110.2	C12B—C13B—H13B	119.6
C10A—C9A—H9AB	110.2	C13B—C14B—C15B	119.8 (3)
H9AA—C9A—H9AB	108.5	C13B—C14B—H14A	120.1
N2A—C10A—C9A	108.2 (3)	C15B—C14B—H14A	120.1
N2A—C10A—H10C	110.1	C16B—C15B—C14B	120.5 (3)
C9A—C10A—H10C	110.1	C16B—C15B—H15A	119.7
N2A—C10A—H10D	110.1	C14B—C15B—H15A	119.7
C9A—C10A—H10D	110.1	O4B—C16B—C15B	125.8 (3)
H10C—C10A—H10D	108.4	O4B—C16B—C18B	113.5 (2)
N2A—C11A—C12A	125.4 (3)	C15B—C16B—C18B	120.7 (3)
N2A—C11A—H11B	117.3	O4B—C17B—H17D	109.5
C12A—C11A—H11B	117.3	O4B—C17B—H17E	109.5
C18A—C12A—C13A	120.5 (3)	H17D—C17B—H17E	109.5
C18A—C12A—C11A	122.1 (3)	O4B—C17B—H17F	109.5
C13A—C12A—C11A	117.5 (3)	H17D—C17B—H17F	109.5
C14A—C13A—C12A	120.5 (3)	H17E—C17B—H17F	109.5
C14A—C13A—H13A	119.7	O2B—C18B—C12B	124.8 (2)
C12A—C13A—H13A	119.7	O2B—C18B—C16B	116.5 (2)
C13A—C14A—C15A	119.5 (3)	C12B—C18B—C16B	118.7 (2)
C13A—C14A—H14B	120.3	O1SB—C1SB—H2SA	109.5
C15A—C14A—H14B	120.3	O1SB—C1SB—H2SB	109.5
C16A—C15A—C14A	121.2 (3)	H2SA—C1SB—H2SB	109.5
C16A—C15A—H15B	119.4	O1SB—C1SB—H2SC	109.5
C14A—C15A—H15B	119.4	H2SA—C1SB—H2SC	109.5
O4A—C16A—C15A	125.9 (3)	H2SB—C1SB—H2SC	109.5
O4A—C16A—C18A	113.8 (3)	O12—Cl1—O13	110.55 (18)
C15A—C16A—C18A	120.3 (3)	O12—Cl1—O14	109.7 (2)
O4A—C17A—H17A	109.5	O13—Cl1—O14	109.61 (19)
O4A—C17A—H17B	109.5	O12—Cl1—O11	109.18 (17)
H17A—C17A—H17B	109.5	O13—Cl1—O11	109.59 (15)
O4A—C17A—H17C	109.5	O14—Cl1—O11	108.18 (18)
H17A—C17A—H17C	109.5	O23—Cl2—O22	109.67 (8)
H17B—C17A—H17C	109.5	O23—Cl2—O21	109.75 (8)
O2A—C18A—C12A	125.2 (3)	O22—Cl2—O21	109.70 (8)
O2A—C18A—C16A	116.7 (3)	O23—Cl2—O24B	77.5 (5)
C12A—C18A—C16A	118.1 (3)	O22—Cl2—O24B	124.7 (6)
O1SA—C1SA—H1SC	109.5	O21—Cl2—O24B	119.1 (6)
O1SA—C1SA—H1SD	109.5	O23—Cl2—O22B	123.6 (7)
H1SC—C1SA—H1SD	109.5	O22—Cl2—O22B	20.8 (5)
O1SA—C1SA—H1SE	109.5	O21—Cl2—O22B	113.3 (7)
H1SC—C1SA—H1SE	109.5	O24B—Cl2—O22B	109.56 (9)
H1SD—C1SA—H1SE	109.5	O23—Cl2—O21B	120.7 (6)
O2B—Mn2—O1B	93.56 (8)	O22—Cl2—O21B	111.6 (7)
O2B—Mn2—N1B	174.04 (10)	O21—Cl2—O21B	14.9 (5)
O1B—Mn2—N1B	91.80 (9)	O24B—Cl2—O21B	109.50 (9)
O2B—Mn2—N2B	91.90 (9)	O22B—Cl2—O21B	109.48 (9)
O1B—Mn2—N2B	174.52 (9)	O23—Cl2—O23B	32.0 (5)
N1B—Mn2—N2B	82.77 (10)	O22—Cl2—O23B	89.8 (6)
O2B—Mn2—O1WB	94.19 (9)	O21—Cl2—O23B	94.8 (5)
O1B—Mn2—O1WB	93.32 (10)	O24B—Cl2—O23B	109.46 (9)
N1B—Mn2—O1WB	88.08 (9)	O22B—Cl2—O23B	109.43 (9)
N2B—Mn2—O1WB	85.80 (9)	O21B—Cl2—O23B	109.38 (9)
O2B—Mn2—O1SB	88.56 (9)	O23—Cl2—O24	109.22 (8)
O1B—Mn2—O1SB	92.06 (10)	O22—Cl2—O24	109.25 (8)
N1B—Mn2—O1SB	88.66 (9)	O21—Cl2—O24	109.23 (8)
N2B—Mn2—O1SB	88.56 (10)	O24B—Cl2—O24	31.8 (5)
O1WB—Mn2—O1SB	173.80 (9)	O22B—Cl2—O24	89.2 (5)
C1B—O1B—Mn2	127.23 (18)	O21B—Cl2—O24	95.1 (5)
C18B—O2B—Mn2	127.95 (17)	O23B—Cl2—O24	141.0 (5)
			
O2A—Mn1—O1A—C1A	−155.3 (2)	O2B—Mn2—O1B—C1B	161.1 (3)
N1A—Mn1—O1A—C1A	23.1 (3)	N1B—Mn2—O1B—C1B	−21.5 (3)
N2A—Mn1—O1A—C1A	−0.7 (10)	N2B—Mn2—O1B—C1B	−13.8 (13)
O1WA—Mn1—O1A—C1A	111.1 (3)	O1WB—Mn2—O1B—C1B	66.7 (3)
O1SA—Mn1—O1A—C1A	−68.4 (3)	O1SB—Mn2—O1B—C1B	−110.2 (3)
O1A—Mn1—O2A—C18A	−177.5 (2)	O1B—Mn2—O2B—C18B	165.1 (2)
N1A—Mn1—O2A—C18A	20.3 (13)	N1B—Mn2—O2B—C18B	11.0 (11)
N2A—Mn1—O2A—C18A	5.5 (2)	N2B—Mn2—O2B—C18B	−15.4 (2)
O1WA—Mn1—O2A—C18A	−82.7 (2)	O1WB—Mn2—O2B—C18B	−101.3 (2)
O1SA—Mn1—O2A—C18A	94.8 (2)	O1SB—Mn2—O2B—C18B	73.1 (2)
O1A—Mn1—O1SA—C1SA	−38.3 (2)	O2B—Mn2—O1SB—C1SB	21.5 (2)
O2A—Mn1—O1SA—C1SA	56.1 (2)	O1B—Mn2—O1SB—C1SB	−72.0 (2)
N1A—Mn1—O1SA—C1SA	−128.9 (2)	N1B—Mn2—O1SB—C1SB	−163.8 (2)
N2A—Mn1—O1SA—C1SA	148.1 (2)	N2B—Mn2—O1SB—C1SB	113.4 (2)
O1WA—Mn1—O1SA—C1SA	154.0 (17)	O1WB—Mn2—O1SB—C1SB	137.9 (7)
O1A—Mn1—N1A—C8A	−16.9 (3)	O2B—Mn2—N1B—C8B	167.7 (9)
O2A—Mn1—N1A—C8A	145.3 (11)	O1B—Mn2—N1B—C8B	13.5 (3)
N2A—Mn1—N1A—C8A	160.3 (3)	N2B—Mn2—N1B—C8B	−165.7 (3)
O1WA—Mn1—N1A—C8A	−111.5 (3)	O1WB—Mn2—N1B—C8B	−79.7 (3)
O1SA—Mn1—N1A—C8A	71.0 (3)	O1SB—Mn2—N1B—C8B	105.5 (3)
O1A—Mn1—N1A—C9A	163.7 (2)	O2B—Mn2—N1B—C9B	−14.5 (11)
O2A—Mn1—N1A—C9A	−34.0 (13)	O1B—Mn2—N1B—C9B	−168.7 (2)
N2A—Mn1—N1A—C9A	−19.1 (2)	N2B—Mn2—N1B—C9B	12.1 (2)
O1WA—Mn1—N1A—C9A	69.2 (2)	O1WB—Mn2—N1B—C9B	98.1 (2)
O1SA—Mn1—N1A—C9A	−108.4 (2)	O1SB—Mn2—N1B—C9B	−76.7 (2)
O1A—Mn1—N2A—C11A	−160.9 (8)	O2B—Mn2—N2B—C11B	10.8 (3)
O2A—Mn1—N2A—C11A	−6.1 (3)	O1B—Mn2—N2B—C11B	−174.2 (10)
N1A—Mn1—N2A—C11A	175.2 (3)	N1B—Mn2—N2B—C11B	−166.5 (3)
O1WA—Mn1—N2A—C11A	87.0 (3)	O1WB—Mn2—N2B—C11B	104.9 (3)
O1SA—Mn1—N2A—C11A	−93.2 (3)	O1SB—Mn2—N2B—C11B	−77.7 (3)
O1A—Mn1—N2A—C10A	18.7 (10)	O2B—Mn2—N2B—C10B	−168.5 (2)
O2A—Mn1—N2A—C10A	173.5 (2)	O1B—Mn2—N2B—C10B	6.5 (12)
N1A—Mn1—N2A—C10A	−5.2 (2)	N1B—Mn2—N2B—C10B	14.2 (2)
O1WA—Mn1—N2A—C10A	−93.4 (2)	O1WB—Mn2—N2B—C10B	−74.4 (2)
O1SA—Mn1—N2A—C10A	86.4 (2)	O1SB—Mn2—N2B—C10B	103.0 (2)
Mn1—O1A—C1A—C2A	163.8 (2)	Mn2—O1B—C1B—C7B	20.1 (4)
Mn1—O1A—C1A—C7A	−17.6 (4)	Mn2—O1B—C1B—C2B	−162.7 (2)
C3A—O3A—C2A—C4A	−1.9 (6)	C3B—O3B—C2B—C4B	10.2 (4)
C3A—O3A—C2A—C1A	177.9 (3)	C3B—O3B—C2B—C1B	−170.3 (3)
O1A—C1A—C2A—O3A	−0.1 (4)	O1B—C1B—C2B—O3B	2.1 (4)
C7A—C1A—C2A—O3A	−178.8 (3)	C7B—C1B—C2B—O3B	179.5 (3)
O1A—C1A—C2A—C4A	179.7 (3)	O1B—C1B—C2B—C4B	−178.4 (3)
C7A—C1A—C2A—C4A	1.0 (5)	C7B—C1B—C2B—C4B	−1.0 (4)
O3A—C2A—C4A—C5A	179.0 (4)	O3B—C2B—C4B—C5B	179.9 (3)
C1A—C2A—C4A—C5A	−0.8 (6)	C1B—C2B—C4B—C5B	0.4 (5)
C2A—C4A—C5A—C6A	0.7 (7)	C2B—C4B—C5B—C6B	0.4 (5)
C4A—C5A—C6A—C7A	−0.8 (6)	C4B—C5B—C6B—C7B	−0.6 (5)
C5A—C6A—C7A—C1A	1.1 (6)	O1B—C1B—C7B—C6B	178.0 (3)
C5A—C6A—C7A—C8A	−176.1 (4)	C2B—C1B—C7B—C6B	0.8 (4)
O1A—C1A—C7A—C6A	−179.8 (3)	O1B—C1B—C7B—C8B	−3.6 (5)
C2A—C1A—C7A—C6A	−1.1 (5)	C2B—C1B—C7B—C8B	179.2 (3)
O1A—C1A—C7A—C8A	−2.6 (5)	C5B—C6B—C7B—C1B	−0.1 (5)
C2A—C1A—C7A—C8A	176.0 (3)	C5B—C6B—C7B—C8B	−178.5 (3)
C9A—N1A—C8A—C7A	−175.9 (3)	C9B—N1B—C8B—C7B	178.5 (3)
Mn1—N1A—C8A—C7A	4.8 (5)	Mn2—N1B—C8B—C7B	−3.9 (4)
C6A—C7A—C8A—N1A	−174.0 (3)	C1B—C7B—C8B—N1B	−4.4 (5)
C1A—C7A—C8A—N1A	8.8 (5)	C6B—C7B—C8B—N1B	174.0 (3)
C8A—N1A—C9A—C10A	−141.3 (3)	C8B—N1B—C9B—C10B	143.8 (3)
Mn1—N1A—C9A—C10A	38.1 (3)	Mn2—N1B—C9B—C10B	−34.1 (3)
C11A—N2A—C10A—C9A	−153.4 (3)	C11B—N2B—C10B—C9B	144.5 (3)
Mn1—N2A—C10A—C9A	26.9 (3)	Mn2—N2B—C10B—C9B	−36.1 (3)
N1A—C9A—C10A—N2A	−41.0 (4)	N1B—C9B—C10B—N2B	44.2 (3)
C10A—N2A—C11A—C12A	−175.2 (3)	C10B—N2B—C11B—C12B	175.6 (3)
Mn1—N2A—C11A—C12A	4.4 (5)	Mn2—N2B—C11B—C12B	−3.7 (4)
N2A—C11A—C12A—C18A	0.4 (5)	N2B—C11B—C12B—C18B	−3.9 (5)
N2A—C11A—C12A—C13A	179.8 (3)	N2B—C11B—C12B—C13B	177.7 (3)
C18A—C12A—C13A—C14A	2.1 (5)	C18B—C12B—C13B—C14B	1.4 (5)
C11A—C12A—C13A—C14A	−177.3 (3)	C11B—C12B—C13B—C14B	179.9 (3)
C12A—C13A—C14A—C15A	−1.3 (5)	C12B—C13B—C14B—C15B	0.1 (5)
C13A—C14A—C15A—C16A	0.4 (5)	C13B—C14B—C15B—C16B	−2.0 (5)
C17A—O4A—C16A—C15A	−8.9 (4)	C17B—O4B—C16B—C15B	0.5 (5)
C17A—O4A—C16A—C18A	171.6 (3)	C17B—O4B—C16B—C18B	−179.7 (3)
C14A—C15A—C16A—O4A	−179.8 (3)	C14B—C15B—C16B—O4B	−177.8 (3)
C14A—C15A—C16A—C18A	−0.4 (5)	C14B—C15B—C16B—C18B	2.4 (5)
Mn1—O2A—C18A—C12A	−2.9 (4)	Mn2—O2B—C18B—C12B	13.1 (4)
Mn1—O2A—C18A—C16A	178.4 (2)	Mn2—O2B—C18B—C16B	−168.1 (2)
C13A—C12A—C18A—O2A	179.2 (3)	C13B—C12B—C18B—O2B	177.8 (3)
C11A—C12A—C18A—O2A	−1.4 (5)	C11B—C12B—C18B—O2B	−0.7 (5)
C13A—C12A—C18A—C16A	−2.0 (4)	C13B—C12B—C18B—C16B	−1.0 (4)
C11A—C12A—C18A—C16A	177.4 (3)	C11B—C12B—C18B—C16B	−179.4 (3)
O4A—C16A—C18A—O2A	−0.5 (4)	O4B—C16B—C18B—O2B	0.5 (4)
C15A—C16A—C18A—O2A	−180.0 (3)	C15B—C16B—C18B—O2B	−179.7 (3)
O4A—C16A—C18A—C12A	−179.3 (3)	O4B—C16B—C18B—C12B	179.3 (3)
C15A—C16A—C18A—C12A	1.2 (4)	C15B—C16B—C18B—C12B	−0.9 (5)

### Hydrogen-bond geometry (Å, °)

**Table:**

*D*—H···*A*	*D*—H	H···*A*	*D*···*A*	*D*—H···*A*
O1SA—H1SA···O11	0.84	1.97	2.788 (3)	165
O1WA—H1W1···O3B	0.80 (2)	2.15 (2)	2.830 (3)	142 (3)
O1WA—H1W1···O1B	0.80 (2)	2.27 (3)	2.964 (3)	145 (3)
O1WA—H1W2···O4B	0.81 (2)	2.19 (2)	2.944 (3)	155 (3)
O1WA—H1W2···O2B	0.81 (2)	2.26 (3)	2.886 (3)	135 (3)
O1SB—H1SB···O21	0.84	2.02	2.737 (4)	143
O1SB—H1SB···O21B	0.84	2.33	3.010 (13)	139
O1SB—H1SB···O23B	0.84	2.50	3.312 (13)	163
O1WB—H1W3···O4A	0.82 (2)	2.23 (2)	2.944 (3)	146 (3)
O1WB—H1W3···O2A	0.82 (2)	2.19 (3)	2.885 (3)	143 (3)
O1WB—H1W4···O1A	0.83 (2)	2.12 (3)	2.868 (3)	151 (3)
O1WB—H1W4···O3A	0.83 (2)	2.36 (2)	2.997 (4)	135 (3)
